# Terlipressin effect on hepatorenal syndrome: Updated meta‐analysis of randomized controlled trials

**DOI:** 10.1002/jgh3.12600

**Published:** 2021-07-01

**Authors:** Mohamed M G Mohamed, Abdul Rauf, Abubakr Adam, Babikir Kheiri, Alexandre Lacasse, Hani El‐Halawany

**Affiliations:** ^1^ Internal Medicine Department SSM Health St. Mary's Hospital‐St. Louis St. Louis Missouri USA; ^2^ Hospital Medicine Department, School of Medicine University of Missouri Colombia Missouri USA; ^3^ Department of Cardiology, Knight Cardiovascular Institute Oregon Health & Science University Portland Oregon USA; ^4^ Department of Gastroenterology, Hepatobiliary and Endoscopy SSM Health St. Mary's Hospital‐St. Louis St. Louis Missouri USA

**Keywords:** albumin, cirrhosis, hepatorenal syndrome, terlipressin

## Abstract

**Background and Aim:**

Hepatorenal syndrome (HRS) is a fatal complication of liver cirrhosis with a limited pharmacological option. Terlipressin is a vasoconstrictor that is approved in many countries but not yet in the United States. This is a meta‐analysis of randomized controlled trials (RCTs) to review terlipressin effect on HRS and the safety profile.

**Methods:**

We searched electronic databases for RCTs comparing terlipressin *versus* placebo in addition to albumin in patients with type 1 or 2 HRS. Primary outcome was HRS reversal. Secondary outcomes were change in serum creatinine (Cr), requirement for renal replacement therapy (RRT) at 30 days of randomization, and 90‐day survival. Risk ratios (RRs) and mean differences (MD) were calculated with 95% confidence intervals (CIs) using a random‐effects model.

**Results:**

We identified eight RCTs with a total of 974 patients, and median follow up of 100 days. Mean age was 55 ± 10 years, 61% were males. Alcoholic liver disease represented 56%. Compared with placebo, terlipressin was associated with a significantly higher likelihood of HRS reversal (RR 2.08; 95% CI [1.51, 2.86], *P* < 0.001), significantly lower serum Cr (MD −0.64; 95% CI (−1.02, −0.27), *P* < 0.001], and a trend toward less RRT requirements (RR 0.61; 95% CI [0.36, 1.02], *P* = 0.06). There was no difference in survival at 90 days between groups (RR 1.09; 95% CI (0.84, 1.43), *P* = 0.52). Major adverse effects (AEs) were gastrointestinal cramps, discomfort, and respiratory distress.

**Conclusion:**

In patients with liver cirrhosis complicated by HRS, terlipressin was associated with significant HRS reversal and decrease in serum Cr. No survival benefit was detected at 90 days.

## Graphical abstract

Hepatorenal syndrome is a fatal complication of hepatic cirrhosis. Terlipressin is a vasopressor with proven benefits in hepatorenal syndrome, with concerns for safety. This meta‐analysis reviewed current evidence and demonstrated terlipressin efficacy and safety.

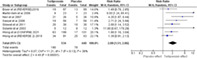

## Introduction

Hepatorenal syndrome (HRS) is a functional renal failure (without intrinsic renal disease) common in patients with decompensated liver cirrhosis.^1^ It is associated with high morbidity and mortality, necessitating hemodialysis, liver transplant, or leading to death in untreated patients.^1^, ^2^

Hypothesized pathophysiology of HRS is altered hemodynamics in splanchnic circulation due to cirrhosis. Visceral and systemic vasodilatation, due to excessive nitric oxide (NO) among other mediators, lead to decreased renal perfusion, with vicious activation of renin–angiotensin–aldosterone system (RAAS).^1^ Based on that, vasoconstrictors have been proposed as therapeutic agents.

Terlipressin is a synthetic vasopressin analogue, with vasoconstrictor properties in splanchnic circulation. It reduces portal hypertension, improves mean arterial pressure and peripheral perfusion, with potential beneficial effects on renal function.^2^ Numerous small observational and experimental trials led to approval of terlipressin in many countries,^3^ including Europe.^4^ Despite that, terlipressin is not yet approved in the United States, due to inconsistent evidence from large, randomized trials, in addition to safety concerns.

Therefore, we conducted an updated meta‐analysis including only randomized controlled trials (RCTs) investigating effects and safety of terlipressin on hepatorenal syndrome in patients with liver cirrhosis.

## Methods

This meta‐analysis was completed according to preferred reporting items for systematic reviews and meta‐analyses (PRISMA) guidelines.

### Study selection and eligibility criteria

Literature search and review was performed by two authors (MM, AR), and disagreements were resolved via a consensus. We searched PubMed/MEDLINE, Embase, and Cochrane databases from inception through March 2021 using the keywords “liver cirrhosis” OR “hepatic cirrhosis” OR “advanced liver disease” OR “advanced hepatic disease” AND “Terlipressin” OR “Vasopressin analogue” AND “hepatorenal syndrome”. Search was restricted to English language. The selection of studies followed a screening of titles and abstracts, and a full‐text review of potentially eligible studies for final determination.

RCT comparing terlipressin to placebo with or without albumin was eligible. We included both type 1 and type 2 HRS. A study must report at least the primary outcome directly or indirectly to be included.

HRS was defined according to international ascites club (ICA) 1996,^5^ or 2007.^6^ Liver cirrhosis was defined based on histology, radiology, or retrospectively if patients presented with decompensation and consistent clinical picture.

We excluded other types of studies (observation, retrospective, and prospective cohort studies). We also excluded studies comparing terlipressin to active treatment (vasoconstrictors), as our aim is to establish efficacy and minimize heterogeneity.

### Outcomes measures

The primary outcome of interest is reversal of HRS (defined as a drop of creatinine [Cr] ≤1.5 mg/dL, or ≥50% baseline, in two occasions within 48 h). Secondary outcomes are the change in serum Cr. from baseline, requirement of renal replacement therapy (RRT) at 30 days of randomization, and 90‐day survival.

We also reported predictors of HRS reversal based on univariate and multivariate regression in all included studies. In addition, we reported terlipressin adverse effects (AEs) and safety features.

### Data analysis

**Figure 1:**
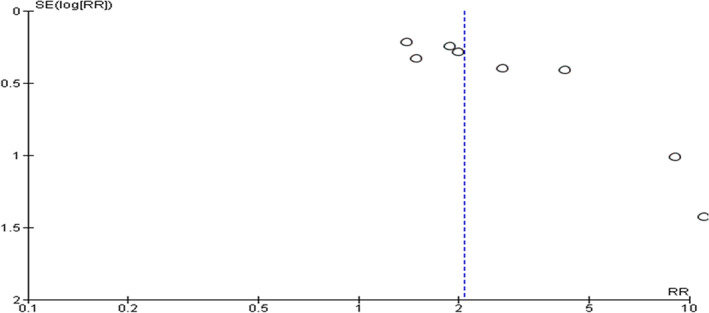
Funnel plot of comparison, reversal of hepatorenal syndrome.

**Figure 2:**
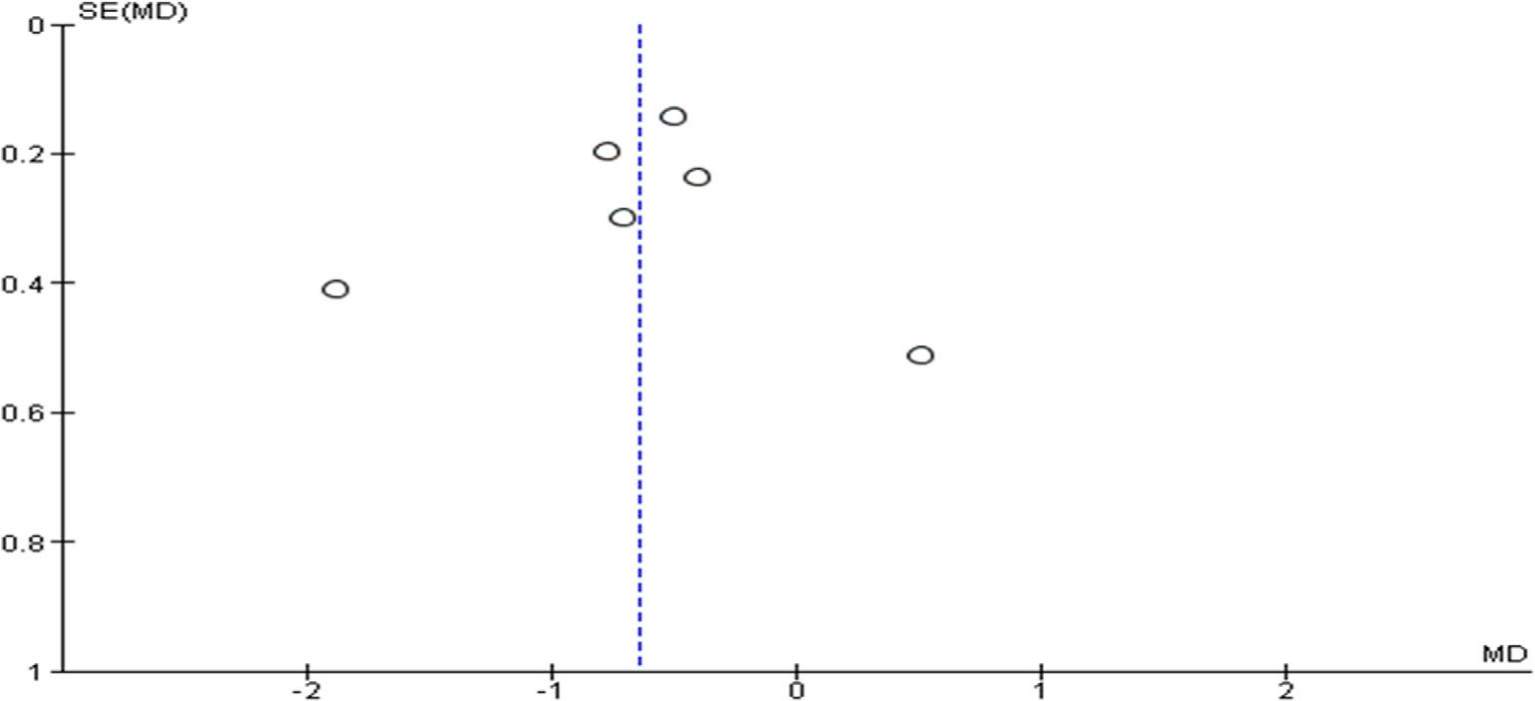
Funnel plot of comparison, change in serum creatinine from baseline.

We calculated risk ratios (RRs) and mean differences (MD) with 95% confidence intervals (CIs) for dichotomous and continuous data, respectively. We assessed heterogeneity using *I* ^2^ statistic. We assessed publication bias using funnel plots in primary outcome (HRS reversal) (Fig. 1), and one secondary outcome (change in serum Cr from baseline) (Fig. 2).

All statistical analyses were done using RevMan manager v5.3 software using a random‐effects model.

## Results

We identified eight RCT (*n* = 974) with a total of 534 patients randomized to terlipressin and 440 patients randomized to placebo (mean age 55 ± 10 years, 61% males) with a median study duration of 14 days and follow up of 100 days.^7^, ^8^, ^9^, ^10^, ^11^, ^12^, ^13^, ^14^ Terlipressin was administered intravenously in a dose of 0.5–2 mg every 6–12 h. Both groups received albumin.

Alcoholic cirrhosis represented 56% of etiologies. Mean model for end‐stage liver disease (MELD) and Child–Pugh scores were 33 ± 6, 10.4 ± 1.8, respectively. Mean arterial pressure (MAP) was 76 ± 11 mmHg, serum sodium was 132 ± 6 mmol/L, serum Cr was 3.6 ± 1.2 mg/dL, serum albumin was 3.4 ± 1 g/dL, total bilirubin was 13 ± 13 mg/dL, and international normalized ratio was 2.3 ± 0.8.

Table 1 shows patients’ baseline characteristics.

**Table 1:** Baseline characteristics of studies included in the meta‐analysis

Study	Country	Age/year	Male	Alcoholic cirrhosis *n* (%)	Mean arterial pressure (mmHg)	Serum Na (mmol/L)	Serum Cr (mg/dL)	Total bilirubin (mg/dL)	Albumin (g/dL)	INR	MELD score	Child–Pugh score
Sample size		M (SD)	*n* (%)		M (SD)							
Boyer et al. (2016) *T* = 97, *C* = 99	North America	55.3 (8.4)	119	103	75.5 (11)	132.1 (6.3)	3.65 (1)	11.65 (11.8)	3.55 (0.7)	2.25 (0.8)	33 (5.9)	10.3 (1.7)
Martín‐Llahí et al. (2008) *T* = 23, *C* = 23	Spain	57(10.6)	29	33	70.5 (11)	126.5 (7.8)	3.85 (2)	14.5 (16)	2.9 (0.65)	X	29 (8.5)	10.5 (2)
Neri et al. (2007) *T* = 26, *C* = 26	Italy	59.5 (3.5)	21	7	85.5 (4)	126 (4.5)	2.85 (1)	X	2.7 (0.3)	X	X	11.35 (0.9)
Sanyal et al. (2008) *T* = 56, *C* = 56	Europe and North America	51.75 (11)	80	58	76.4 (12.5)	131.5 (7)	3.9 (1.7)	15.4 (14.3)	2.75 (0.8)	2.3 (1)	33.4 (6.15)	11.5 (1.85)
Silawat et al. (2011) *T* = 30, *C* = 30	Pakistan	X	X	X	68 (15)	133 (4.27)	3.01 (1.25)	X	2.45 (0.67)	X	X	X
Solanki et al. (2003) *T* = 12, *C* = 12	India	51.5 (5)	17	X	75 (1)	X	2.6 (0.15)	7.1 (1.2)	3.1 (0.1)	X	X	X
Wong et al. (2020) *T* = 91, *C* = 93	North America	55.6 (8.4)	112	98	75.7 (11.4)	132.4 (6)	3.6 (1)	11.4 (11.6)	3.5 (0.7)	2.3 (0.8)	33 (5.76)	10.4 (1.75)
Wong et al. (2021) *T* = 199, *C* = 101	North America	53.8 (11.5)	179	201	78.3 (11.3)	133 (5.5)	3.5 (1)	13.7 (14)	3.8 (1.6)	x	32.8 (6.5)	10 (1.86)
Totals: 974 *T* = 534, *C* = 440		55 (10)	540 (61)	500 (56)	76 (11)	132 (6)	3.6 (1.2)	13 (13)	3.4 (1)	2.3 (0.8)	33 (6)	10.4 (1.8)

All values are mean (SD) or number (%).

C, control; Cr, creatinine; INR, international normalized ratio; MELD, mean model for end‐stage liver disease; T, Terlipressin; X, unavailable data.

**Figure 3:**
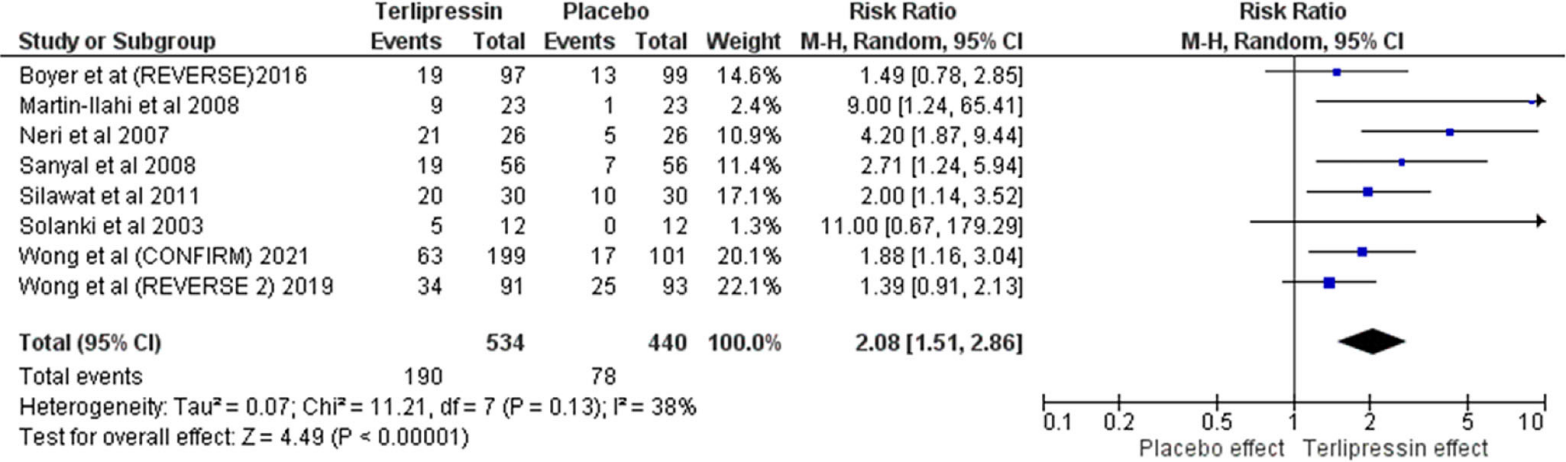
Primary outcome; reversal of hepatorenal syndrome.

**Figure 4:**
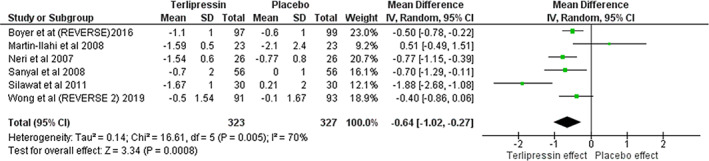
Secondary outcome; change in baseline serum creatinine (mg/dL).

**Figure 5:**
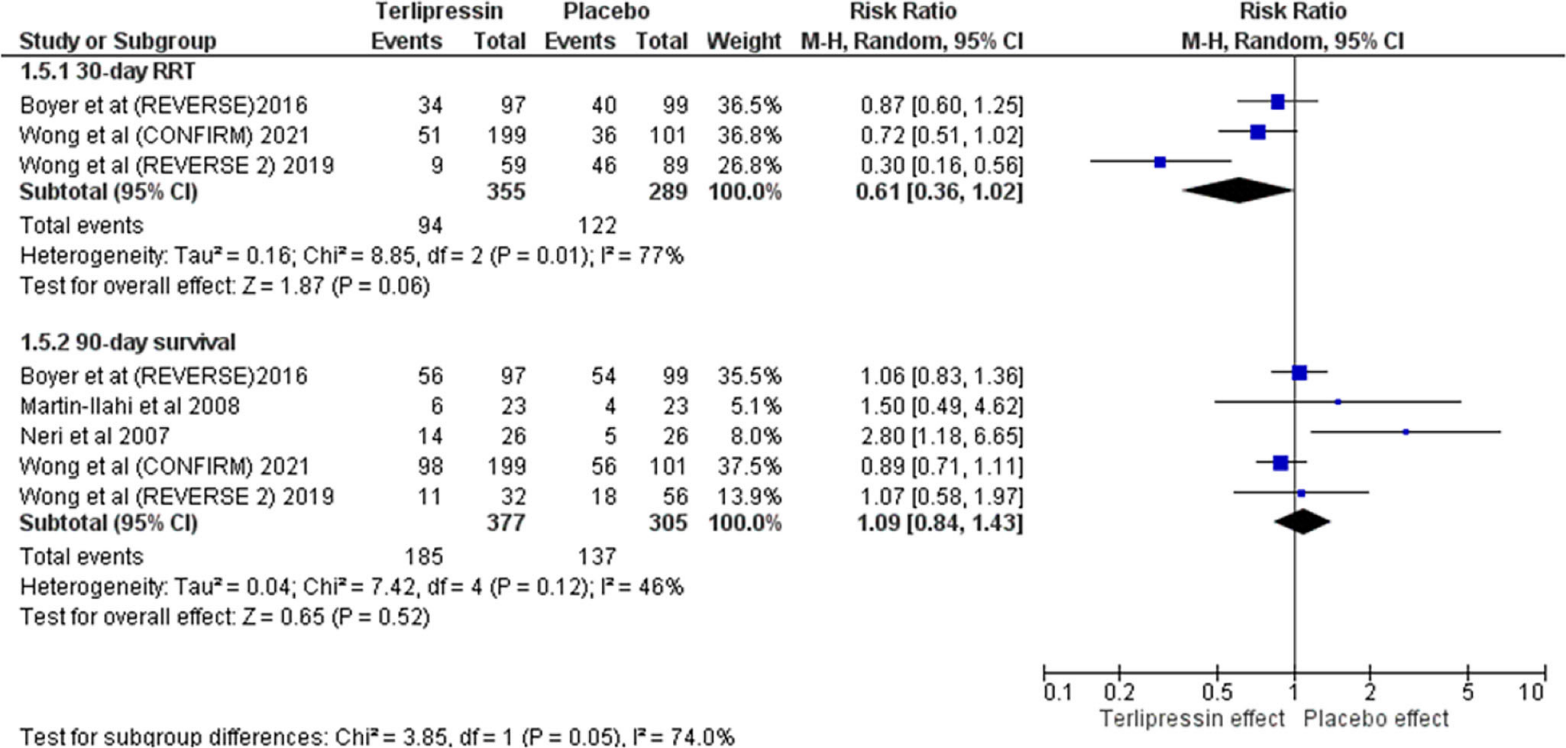
(a, b) Secondary outcomes; requirement of renal replacement therapy at 30‐day from randomization, and 90‐day survival.

Compared with placebo, terlipressin was associated with a significant higher likelihood of HRS reversal (RR 2.08; 95% CI [1.51, 2.86], *P* < 0.001] (Fig. 3). Terlipressin was also associated with a significant decrease in baseline serum Cr (MD −0.64; 95% CI [−1.02, −0.27], *P* < 0.001) (Fig. 4). Terlipressin showed a trend toward decreased requirement for RRT at 30 days but was not statistically significant (RR 0.61; 95% CI [0.36, 1.02], *P* = 0.06) (Fig. 5a). Terlipressin showed no survival benefit compared with placebo at 90 days (RR 1.09; 95% CI [0.84, 1.43], *P* = 0.52) (Fig. 3).

Predictors of HRS reversal based on regression models were younger age, low MELD and Child–Pugh scores, low baseline serum Cr., and total bilirubin levels, and non‐alcohol hepatitis (Table 2).

**Table 2:** Predictors of hepatorenal syndrome (HRS) reversal in regression models

Study	Predictors of HRS reversal
Boyer et al. (2016)	Low MELD (*P* < 0.1) Low total bilirubin (*P* = 0.1) Low serum creatinine (*P* = 0.05)
Martin et al. (2008)	Multi: serum creatinine (*P* < 0.001) Urine volume (*P* = 0.005) White Blood Cells count (*P* = 0.001)
Neri et al. (2007)	Younger age (*P* < 0.001) Child–Pugh score ≤12 (*P* < 0.05)
Sanyal et al. (2008)	Serum creatinine (*P* = 0.021) MELD score (*P* = 0.017)
Silawat et al. (2011)	NA
Solanki et al. (2003)	NA
Wong et al. (2020)	Non‐alcohol hepatitis (*P* = 0.04) Lower serum creatinine (*P* = 0.0003)
Wong et al. (2021)	NA

MELD, mean model for end‐stage liver disease; NA, not available.

Terlipressin was more associated with nausea, abdominal pain, diarrhea, cardiac dysrhythmia, ischemia, and pulmonary edema than placebo.

## Discussion

This meta‐analysis showed that the use of terlipressin with albumin in patients with liver cirrhosis and HRS is superior to placebo and albumin alone, regarding likelihood of HRS reversal, decrease in baseline serum Cr, with a trend toward less requirement of RRT at 30 days. Terlipressin showed no survival benefit at 90 days.

These findings are consistent with existing literature.^15^, ^16^ Terlipressin, as a splanchnic vasoconstrictor, optimizes hemodynamics and improves renal perfusion in patients with cirrhosis. This translates as a beneficial effect on renal function. Reversal of HRS is an important outcome in patients with HRS, as the prognosis is poor in untreated patients.^17^

Systemic inflammatory response syndrome (SIRS) also probably plays a role in HRS.^18^ Although our study was not designed or powered to report the effect of terlipressin on SIRS directly, it has been postulated that terlipressin, by its vasoconstrictor effect on portal circulation, protects from bacterial translocation, endotoxemia, and subsequent pro‐inflammatory cytokines. This effect probably facilitates and augments the response of terlipressin in patients with decompensated liver cirrhosis.^19^

A meta‐analysis of seven trials (*n* = 345) showed a significant correlation between decrease in serum Cr with vasoconstrictor treatment and reduction in mortality.^20^ Notably, various combinations of terlipressin, octreotide, midodrine, dopamine, norepinephrine, and placebo were included. In our analysis, we only compared terlipressin with placebo, allowing albumin in both groups, to establish efficacy and minimize heterogeneity.

Numerous studies reported advantage of terlipressin over other vasoconstrictors. One RCT compared terlipressin *versus* octreotide and showed that terlipressin led to more HRS reversal than octreotide (55% *vs* 20%, *P* = 0.01).^21^ Another RCT compared terlipressin *versus* octreotide and midodrine. This study showed that terlipressin and albumin were more effective in reversing renal failure (55.5% *vs* 4.8%, *P* < 0.001) and improving renal function in HRS (70.4% *vs* 28.6%, *P* = 0.01).^22^ Another study compared terlipressin *versus* norepinephrine in acute‐on‐chronic liver failure patients with HRS, it showed that terlipressin was superior to norepinephrine in HRS reversal (40% *vs* 16.7%, *P* = 0.004).^23^

Although terlipressin improved kidney function and lead to significant HRS reversal, there was no survival benefit at 90 days compared with placebo. This is probably due to the high risk of other fatal complications unrelated to HRS, which necessitate liver transplantation.^24^ Terlipressin only optimizes hemodynamics and subsequently improves renal function but does not eliminate the disturbed milieu in hepatic cirrhosis. Cirrhotic patients have inherently high mortality and suffer from other comorbidities that might culminate in death even after reversal of HRS. In addition, our study population have advanced hepatic disease on enrolment with mean MELD score of 33 ± 6.

Lower disease stage at presentation (measured by MELD, or Child–Pugh scores), in addition to younger age, lower baseline Cr, and bilirubin were predictors of HRS reversal and better outcome. This was expected, as earlier presentation in younger patients with less comorbid conditions and more preserved hepatic and renal function usually lead to favorable disease course.

Terlipressin is well tolerated, with mostly gastrointestinal AE (nausea, diarrhea, abdominal cramps). Respiratory distress and failure, which were noted more with terlipressin, are probably due to pulmonary edema secondary to increase in preload (venoconstriction) and afterload (arterio‐constriction).^25^, ^26^ Cardiac dysrhythmias and ischemic events, as expected, were reported more with terlipressin than placebo group. Therefore, terlipressin use should be limited to patients with advanced liver disease and should be used with caution in patients with limited cardiopulmonary reserve.

Our study is the most updated study including the recently published RCT by Wong et al.,^7^ we also included only RCT to exclude inherent observational studies biases and weaknesses. On the other hand, our study has some limitations. There is a heterogeneity regarding dosing and escalation protocol of terlipressin between studies. The same applies to albumin. Also, females are underrepresented in this analysis. Moreover, only three RCTs reported RRT outcome.

In conclusion, in patients with liver cirrhosis complicated by HRS, the use of terlipressin with albumin is associated with higher likelihood of HRS reversal, and decrease in serum Cr., than albumin alone. Terlipressin might cause gastrointestinal AE and worsening respiratory distress in certain patients. Terlipressin showed no survival benefit at 90 days.
